# Advancing the evidence base in cancer: psychosocial multicenter trials

**DOI:** 10.1186/1745-6215-13-171

**Published:** 2012-09-19

**Authors:** Robert Sanson-Fisher, Lisa Mackenzie, Phyllis Butow, Nicole Rankin, Christine Paul

**Affiliations:** 1 The University of Newcastle, University Drive, Callaghan, NSW, 2308, Australia; 2 Hunter Medical Research Institute, Newcastle, Australia; 3 The University of Sydney, Sydney, NSW, 2006, Australia; 4 School of Medicine and Public Health, Faculty of Health, University of Newcastle, University Drive, Callaghan, NSW, 2308, Australia

**Keywords:** Cancer, Oncology, Multicenter trials, Psychosocial aspects, Intervention studies

## Abstract

**Background:**

The diagnosis and treatment of cancer is associated with significant distress and psychosocial morbidity. Although psychosocial interventions have been developed in an attempt to improve psychosocial outcomes in cancer patients and survivors, there is continued debate about whether there is adequate high-level evidence to establish the effectiveness of these interventions. The evidence base is limited as a result of numerous challenges faced by those attempting to conduct psychosocial intervention trials within the health system. Barriers include insufficient participant recruitment, difficulty generalizing from single-trial studies, difficulty in building and managing research teams with multidisciplinary expertise, lack of research design expertise and a lack of incentives for researchers conducting intervention research. To strengthen the evidence base, more intervention studies employing methodologically rigorous research designs are necessary.

**Methods:**

In order to advance the evidence base of interventions designed to improve psychosocial outcomes for cancer patients and survivors, we propose the formation of a collaborative trials group that conducts multicenter trials to test the effectiveness of such interventions.

**Results:**

Establishment of such a group would improve the quality of the evidence base in psychosocial research in cancer patients, by increasing support for conducting intervention research and providing intervention research training opportunities. A multidisciplinary collaborative group conducting multicenter trials would have the capacity to overcome many of the barriers that currently exist.

**Conclusions:**

A stronger evidence base is necessary to identify effective psychosocial interventions for cancer patients. The proposed formation of a psycho-oncology collaborative trials group that conducts multicenter trials to test the effectiveness of psychosocial interventions would assist in achieving this outcome.

## Background

The diagnosis and treatment of cancer causes significant psychosocial distress in cancer patients and survivors across the disease trajectory [1]. Despite a clear need for high quality research into what constitutes effective psychosocial interventions for cancer patients [2], reviews of the literature reveal a field primarily focused on descriptive research [3,4]. Although descriptive research explores the epidemiological aspects of quality of life for cancer patients, it is unable to establish causation. Health policy aims to be guided by high quality empirical evidence of intervention effectiveness. The strongest level of evidence is produced when randomized controlled trials (RCTs) demonstrate repeated effects [5]. Once effective interventions are demonstrated, there is room for experimental exploration of dissemination methods so that Level 1 evidence interventions (with multiple RCTs having demonstrated effectiveness) become a routine component of patient care [5].

In recent years there have been efforts to test psychosocial interventions and implement clinical practice guidelines for the management of acute psychosocial distress in cancer patients and their families [6]. While there is some evidence that these interventions increase well-being, improve coping and adjustment, and reduce distress [7-11] there are also suggestions that psychosocial interventions are ineffective at reducing distress [12-14] and debate about their acceptability to patients [9,12-14].

Conflicting views about the available body of psychosocial intervention research may largely be explained by limitations of design and content focus [15,16]. Jacobsen and Jim’s [15] overview of reviews of psychosocial interventions for anxiety and depression indicated that shortcomings related to poor research quality and variation in methods have been consistently identified. Common design deficiencies include lack of a control group, non-randomization, small or biased samples and non-validated outcome measures, each of which can compromise the validity of study findings. As a consequence, specific intervention effects and treatment recommendations have been difficult to delineate [16]. This is particularly so for patients with certain demographic, disease and treatment characteristics such as men and those with less common cancers or advanced disease [17]. In order to provide practice-relevant treatment recommendations for psychosocial cancer care, there remains a need for methodologically sound psychosocial intervention studies across a range of patient groups [17].

In this article, we discuss some of the barriers experienced by researchers attempting to conduct psychosocial intervention trials in the health system and outline how the formation of a multicenter trial collaborative group that conducts psychosocial intervention trials could significantly contribute to the evidence base.

### Barriers to developing the knowledge base for evidence-based practice

The shortcomings of the psychosocial intervention literature are not surprising given the many challenges researchers face in mounting a rigorous intervention study within a complex health system. Potential barriers to such research include recruitment and design challenges, scarcity of the required multidisciplinary skills, financial constraints and health system challenges.

#### Recruitment challenges

Insufficient participant recruitment into RCTs presents a constant challenge to researchers [18], as this limits statistical power to detect treatment effects. Additionally, external validity may be threatened due to small samples being less representative of the population in which the intervention may be used [19]. Randomization of cancer patients to different treatment groups as required by RCT designs may present additional difficulties in participant retention [20].

A number of factors may contribute to under-recruitment. Professional and familial gate-keeping are a common difficulty, meaning options for recruitment sites and access to some patient and carer populations may be limited [20]. Funding limitations and the need for researcher proximity also may limit recruitment to a single treatment center source (possibly where the researcher is based or has a reputation). Recruitment via a single treatment center tends to be slow, particularly when focused on a population defined by diagnosis or disease stage [21]. Rapid recruitment of an adequate sample size can be even more challenging when carrying out investigations in rural and regional centers [22]. It is likely that limited access to treatment centers has guided research conception, limiting the number of intervention studies proposed and conducted.

#### Difficulties comparing studies using different outcomes and outcomes measures

There has been concern and debate relating to which psychological constructs and measures are the most likely to reflect clinically meaningful changes following different styles of psychosocial interventions [23]. Interventions have evaluated impacts on combinations of psychosocial constructs, including (but not limited to) anxiety, depression, distress, Post-Traumatic Stress Disorder (PTSD) symptoms, quality of life, unmet needs, fear of cancer recurrence and satisfaction with care [24]. Within these constructs, varied measures have been used. For instance, there has been controversy about which psychometric tests are most appropriate for use as outcome measures in psycho-oncology research [25]. Three of the most commonly used assessment alternatives include the Hospital Anxiety and Depression Scale (HADS) [26], the Depression Anxiety Stress Scale (DASS) [27], and the more recently developed ultra short screening tools such as the Distress Thermometer (DT) [28]. This use of non-uniform constructs and measures of the prevalence and predictors of psychosocial morbidity has led to difficulties in interpreting the available research data due to an inability to collapse results across studies.

#### Appropriateness of research design

The Consolidated Standards of Reporting Trials (CONSORT) group has developed guidelines to increase research ability to adhere to and assess research against a ‘gold standard’ research design and methodology. Although the RCT has been considered the ‘gold standard’ research design, alternative designs have been encouraged for assessing population- and systems-based interventions, where randomization is impractical, costly or unethical [29,30]. A failure of widespread adherence of psychosocial trials to the CONSORT guidelines has been identified by reviews of psychosocial interventions for cancer patients [7,14], and for adolescents and young adults [31] with cancer. Although RCTs are useful for safeguarding internal validity in interventions where the individual is the unit of randomization, when the unit of randomization is the treatment center this design becomes more complex [30]. In selecting the most appropriate study designs, tradeoffs between the RCT and alternative designs, including cohort and case-control, simple pre-post, and multiple baseline [32] designs, may have been justifiable. However, the decision to use designs such as pre-post designs without a control group weakens the validity of study findings and has contributed to the current lack of an evidence base for treatment recommendations. Issues of design are crucial and underscore the need for strong multidisciplinary teams to drive intervention research.

In addition to adherence to CONSORT guidelines, since 2005, the International Committee of Medical Journal Editors (ICMJE) and some grant bodies require prospective registration of trials with the relevant clinical trials registry, such as the Australian and New Zealand Clinical Trials Registry (ANZCTR). However, trial registration remains a voluntary process for researchers outside these requirements. For example, it has been reported that there were low levels of trial registration and outcomes measure definition in manuscripts published in behavioral science journals between 2008 and 2009 [33]. If this finding extends to psycho-oncology, there may be implications for publication bias that may leave the psycho-oncology research community less informed about appropriateness of outcomes measures for assessing particular interventions.

#### Availability of multidisciplinary research skills

A rigorous psychosocial intervention study requires a well-coordinated and committed multidisciplinary team with a range of clinical, research and stakeholder-related expertise. It also involves the application of appropriate study design, knowledge of methodological criteria for outcome measures, ability to interpret results using complex and appropriate statistical analyses and knowledge of ethical procedures and safeguards. In intervention trials undertaken in a single center it may be both challenging and costly to gather and fund relevant expertise across fields of clinical care, biostatistics, health economics, research design and psychometrics.

#### Financial barriers to undertaking intervention research

The relative costs, necessary resources and time required to conduct intervention research is also a likely contributing factor to the paucity of multicenter trials in psycho-oncology. Descriptive studies may be easier to initiate because they create less resource demand while enabling journal publications. Additionally, positive results from intervention trials are by no means guaranteed, and there is a well-recognized bias against publishing negative findings [7]. Such factors may contribute to a view that intervention research is financially and professionally risky.

#### Health system challenges

Large scale intervention studies may require changes to complex, overburdened health systems. It may be difficult for small groups of researchers to persevere over long time periods to sufficiently implement changes required for intervention delivery. Many health systems are experiencing over-stretched resources, which limits the ability of staff to attend training and undertake new or extra tasks unless the organization is committed to providing the necessary extra resources for even small changes to current practice.

#### Lack of agreement about critical research questions

Ensuring advancement in the evidence base requires agreement on the number of intervention studies to be undertaken within a defined time period, on which treatment options most need to be tested, and on which outcomes or constructs the effects will be assessed. Achieving agreement in each of these areas may be a difficult task due to specific areas of interest of some researchers or practitioners. Additionally, there is a need for consensus on the most important research questions to be asked in groups of patients with rare cancer types to reduce the research burden of these groups. There is increasing recognition of the need not only to test the efficacy of a treatment in relation to a placebo or usual care but also to compare the differential effectiveness of available best options. This approach known as comparative effectiveness research has been argued by the Institute of Medicine to be a necessary process in order to improve health outcomes for specific patient groups [34].

## Methods

### Increasing evidence by increasing intervention trials: a psycho-oncology multicenter trial collaborative

To encourage a greater capacity to produce high quality evidence of the effectiveness of specific psychosocial interventions, the authors propose the concept of multicenter psychosocial collaborative trials groups. Such a collaborative trials group would overcome many of the aforementioned barriers, by prioritizing research orientation toward intervention research [35] and building or enhancing collaborative environments/relationships between researchers and clinicians to overcome the barriers described above. In addition, such an approach can assist in increasing the robustness of the evidence base by limiting publication bias and increasing capacity to conduct psychosocial research.

Clinical trials in medicine have used multicenter trials to increase methodologically rigorous intervention research, and consequently improve the evidence base. The benefits of multicenter trials include increased access to participants, the possibility of inclusion of a wider range of population groups, access to different geographic locations, and the ability to compare results across centers, all of which increase study generalizability. Multicenter trials provide the means for testing intervention efficacy between population groups with differing demographic backgrounds. For instance, clinical trials conducted by the International Breast Cancer Study Group (IBCSG) include institutions and cooperative groups from multiple countries. This widespread access to a participant pool has allowed the investigation of treatments that are tailored to specific population groups, for instance older women with endocrine non-responsive breast cancer or pre-menopausal women with endocrine responsive breast cancer [36]. Similarly, the Cancer and Leukemia Group B (CALGB) psycho-oncology committee in the US has conducted quality of life studies as part of CALGB clinical trials focusing on the long-term adjustment of cancer survivors and the effect of treatment on quality of life over a 25 year period [37]. By adopting the collaborative process, a psycho-oncology multicenter trial collaborative can enable increased access to resources and encourage methodologically rigorous psychosocial research.

## Results and discussion

### How would a multicenter trial collaborative address the identified barriers and increase intervention research?

#### Addressing recruitment issues

A collaborative multicenter trials group would enable the simultaneous recruitment of participants from a broad range of collaborative centers, reducing costs and problems associated with extended recruitment time and allowing research into specific patient populations. In Australia, similar models exist within tumor - (Australian Gastro Intestinal Trials Group, http://www.gicancertrials.org.au) and treatment-specific clinical trials groups (for example, Trans-Tasman Radiation Oncology Group, http://www.trog.com.au).

In such models, each participating treatment center enters into an agreement to take part in a specified number of intervention trials over a defined period in order to contribute to the subject pool for different projects. In applying such a model in psycho-oncology research, the profile of psychosocial research might be given a greater priority within the treatment center, and competition for participants within the center could be reduced. This influence over key decision makers is expected to increase recruitment to psychosocial trials [38]. The collaborative multicenter trials group could apply strategies to increase family and health care provider engagement in the research process, including increasing awareness of the voluntary nature of research involvement and the patients’ ability to withdraw from research studies at any stage. This may help to reduce professional and familial gate-keeping to appropriate levels [20].

#### Core measurement tools to increase replicability of results

A collaborative trials group could recommend a core set of standardized instruments with proven reliability and validity. This would accelerate the acquisition of data which can be subjected to meta-analyses, allowing much stronger conclusions to be drawn regarding intervention outcomes. In addition, the necessary training to ensure consistency in intervention delivery across treatment centers and by individual clinicians may be easier to monitor if appropriate resources are available from the collaborative trials group.

#### Ensuring appropriateness and rigor in research design

Methodologically stringent designs could be ensured within a multicenter trial group by having access to appropriate multi-disciplinary intervention research expertise. An established scientific advisory group would provide careful scrutiny of ideas and methodology as well as advice on topic selection and core staff involvement. State-of-the-art therapies could be consistently delivered to patients in different treatment centers. Good Clinical Research Practice (GCRP) accreditation would recognize the collaborative and the involved treatment centers for increasing skill levels through regional and international workshops on research methods in psychosocial oncology in order to raise research quality within the field [39]. A collaborative multicenter group would provide the expertise for reviewing intervention research protocols to ensure that they would meet set standards of intervention research methodology.

#### Availability of multidisciplinary expertise

Multidisciplinary membership of the collaborative trials group and enhanced communication through the group’s channels using on-line technologies, would increase the likelihood of forming teams of experienced investigators with the required expertise to design and implement complex research protocols.

For each study, a core team should be comprised of researchers from disciplines including behavioral science, psychology, biostatistics and health economics, as well as relevant clinicians with expertise in the development, implementation and evaluation of intervention research. The core team structure would comprise different individuals for each collaborative project, given that researchers and clinicians alike will have particular interests and expertise with various interventions and specific patient populations. Where gaps in expertise are apparent, staff employed by the collaborative (such as the executive officer) can help to identify individuals from the group’s membership to join the team. Interaction with colleagues can be facilitated by electronic communication where team members are based in different locations, as well as annual workshops to provide additional opportunities for robust discussion of protocols. Additionally, increased collaboration between clinical and research staff is likely to result in more clinically relevant research.

#### Ability to agree on research priorities

A multicenter trials group would be in a position to establish agreed priorities for research. The Institute of Medicine approach involved a consultative process to establish 100 top priorities for health research [34]. It would seem reasonable that such a process could also be done within a psychosocial collaborative multicenter trials group. Such a process would reduce potential for bias and influence from individual researchers toward their particular areas of interest. Additionally, this process may also be useful for ensuring that small population groups will not be overburdened by responsiveness to the priority setting process. This process could be undertaken on a biannual or annual basis thereby leading to agreement on the most important areas which need to be addressed. Factors such as the prevalence of the condition to be studied, the burden of illness and the probability of being able to answer the question within an acceptable time frame could guide final decisions about research priorities.

#### Increased ability to attract research grants

Under the collaborative model, funding proposals would have an increased likelihood of including appropriate expertise, clear structures and procedures for ensuring excellence and rigor, and recognized standing and credibility. Therefore, proposals coming from such a group would maximize the likelihood of attracting research funding over time. By demonstrating an international standard of high quality research, the multicenter group may attract grants from international funding bodies. Strict protocols within the national body could be put into place regarding the submission of proposed study designs to an advisory board responsible for approving appropriate designs and advising on modifications to inadequate designs. It would be ensured that panel members of the collaborative had a strong track record in methodologically rigorous intervention research. It may also be of benefit for the group to work with funding bodies to ensure that additional funding allocations are provided for well-designed intervention studies.

#### Addressing health system challenges to increase external validity and specificity of results

The ability to recruit a large population-based sample through multicenter collaboration may produce generalizable results, as well as the ability to test cost-benefit ratios and intervention effectiveness in realistic clinical settings. The ability to recruit large sample sizes from specified populations receiving specific interventions, will lead to quality, generalizable research data regarding the impact of such interventions. A multicenter trial group would allow studies to be conducted which use treatment centers, rather than individuals, as the unit of analysis. This would enable the evaluation of intervention effectiveness across groups [3], which should attract attention from policy writers [40]*.*

#### Minimizing publication bias

In order to overcome publication biases against publishing negative findings, all intervention studies proposed by the multicenter trials group would be included within a registry, and both positive and negative findings (published and unpublished) would be available via this means [41]. A number of countries including Australia now have clinical trials registries, and the National Health and Medical Research Council (NHMRC) has mandated registration for NHMRC-funded trials.

#### Increased research capacity

A multicenter collaborative trials group has the potential to build research capacity due to having in place a number of strategies to attract and train new and early career researchers in intervention research, as well as attracting researchers who have a strong track record in intervention research. Options to develop this capacity include providing master classes to develop high quality research proposals [42] that enable the provision of both local and international expertise to researchers-in-training, and including junior researchers on large grants. It is hoped that these initiatives would foster a spirit of collaboration and develop capacity for high quality psychosocial intervention research.

#### Increasing focus on recruitment to trials

There are a number of collaborative research groups already operating in Australia and internationally. The National Comprehensive Cancer Network (NCCN) is an alliance of cancer centers in the US which aims to work together to develop treatment guidelines for most cancers [43]. Additionally, the Canadian Association for Psychosocial Oncology has developed a set of standards for psychosocial support services, called National Psychosocial Oncology Standards [44]. The Psycho-Oncology Co-operative Research Group (PoCoG) was formed in Australia in 2005 to foster multicenter collaborative Psycho-Oncology trials, and is the only organization of which we are aware with this mandate internationally [45]. As yet these groups do not appear to have increased patient recruitment to trials in the manner of the tumor- and clinical-treatment-specific trial groupings. A network of collaborating centers is required involving ongoing commitment from a range of multi-disciplinary groupings with a strong intervention trial focus.

### How would the success of a collaborative trials group be determined?

A number of key outcomes can be used to assess the success of research collaboratives, with the key consideration being the likelihood of producing evidence regarding improving patient outcomes. Other measures of success might include:

• A substantial increase in the proportion of all cancer patients who are recruited into trials of psychosocial interventions.

• Process measures such as length of collaborations, the number of people and centres which are involved and its visibility in the research and general community.

• Contribution to high order evidence (i.e. Level I or Level II evidence). Output might be classified in terms of the number of studies or papers contributing to each level of evidence.

• Adoption of evidence-based practice. The very end-point of success would be the ability of the cooperative to increase the adoption of evidence-based care. Given that care concepts which are not grounded in evidence have the potential to be either harmful or wasteful, the degree to which the group can facilitate a focus on evidence-based practice is the benchmark of its success in helping patients.

### Potential problems of a psycho-oncology collaborative trials group designed to increase intervention research

There is a degree of risk associated with establishing collaborative trials groups. Foremost, ownership of intellectual property must be well protected. Individual researchers may fear the loss of intellectual ownership and hence be unwilling to offer ideas and work with others in the collaborative trials group. Overt and shared views regarding optimal research teams and advisory groups need to be negotiated. Additional potential problems may be associated with differing interpretations of methodological rigor, the need for well-developed protocols to ensure effective trial management, and the question of the group’s sustainability based on external research grants. It may also be difficult to ensure that the ratio of intervention to descriptive research is maintained at a defined acceptable level. Lastly, time taken to review submitted protocols may lead to perceived unacceptable delays, and potential loss of funding opportunities. It must be acknowledged that for the individual researcher there are potential risks and costs associated with such a collaborative. Transparent processes for dealing with concerns and attempting to achieve equitable outcomes for all involved are crucial to maintaining goodwill within such a collaborative.

However it should be considered by potential members of the collaborative that their membership may lead to a range of professional gains. Involvement in the collaborative may present increased opportunities to earn authorship, to be involved in grant writing and, as an investigator, to take on roles as clinical change champions and to work with and learn from a multidisciplinary approach to psychosocial research. Models from successful medical and clinical trials groups may help to guide this process. Clearly defined authorship guidelines for papers and research grants could be initially administered by senior collaborative members, who could allocate topics to a junior or senior principal author and co-authors during the early research priority setting stage. This should help to ensure explicit understandings about authorship between collaborative members. This process should also provide opportunities for capacity building and mentorship. Principal authors should then ensure that collaborative findings are authored by the investigators who collaborate on the work. All contributors, financial support, material support and research participants should be acknowledged, along with the collaboration. Senior members would be responsible for ensuring publication of collaborative research.

## Conclusions

To strengthen the evidence base regarding the effectiveness of psychosocial interventions for cancer, more intervention studies employing methodologically rigorous research designs are required. One method by which this might be achieved is the formation of a Psycho-Oncology collaborative trials group, which undertakes multicenter trials to test the effectiveness of psychosocial interventions. This model allows a broad range of expertise to be included within each research protocol, while still being cost effective and flexible. Research participants could come from many different treatment centers, allowing rapid recruitment of the target sample size and specified population. This would also enable the evaluation of the effectiveness of specific interventions, and the generalization of this to a nationwide population.

The proposed multicenter trial model presents a challenge to researchers, as well as great possibilities for improving the standard of research in the area of psychosocial interventions for cancer care. A network of Collaborating Centers across multiple treatment centers has the potential to overcome many of the current barriers, and could make a significant contribution to the current evidence base that evaluates the effectiveness of psychosocial interventions in cancer care. Such a network is likely best formed nationally, but the potential for linkages and eventually an international network is great and could lead to greater advances particularly in rare cancers or diagnostic groups.

## Abbreviations

CALGB: Cancer and Leukemia Group B; CONSORT: Consolidated Standards of Reporting Trials; DASS: Depression Anxiety Stress Scale; DT: Distress Thermometer; GCRP: Good Clinical Research Practice; HADS: Hospital Anxiety and Depression Scale; IBCSG: International Breast Cancer Study Group; NCCN: National Comprehensive Cancer Network; NHMRC: National Health and Medical Research Council; PoCoG: Psycho-Oncology Co-operative Research Group; RCT: Randomized Controlled Trial; PTSD: Post-Traumatic Stress Disorder.

## Competing interests

The authors declare that they have no competing interests.

## Authors’ contributions

All authors contributed to the conceptualization and drafting of the manuscript. All authors read and approved the final manuscript.
